# Early Postoperative Effects of Cataract Surgery on Anterior Segment Parameters in Primary Open-Angle Glaucoma and Pseudoexfoliation Glaucoma

**DOI:** 10.4274/tjo.92604

**Published:** 2016-06-06

**Authors:** Ufuk Elgin, Emine Şen, Tülay Şimşek, Kemal Tekin, Pelin Yılmazbaş

**Affiliations:** 1 Ulucanlar Eye Research and Training Hospital, Ophthalmology Clinic, Ankara, Turkey; 2 Osmangazi University Faculty of Medicine, Department of Ophthalmology, Eskişehir, Turkey

**Keywords:** Cataract, primary open-angle glaucoma, pseudoexfoliation glaucoma, optical biometer, anterior segment parameters

## Abstract

**Objectives:**

To compare the effect of cataract surgery on anterior segment parameters measured by optical biometry in primary open-angle glaucoma (POAG) and pseudoexfoliation glaucoma (PXG).

**Materials and Methods:**

Twenty-five eyes of 25 patients with POAG and 29 eyes of 29 patients with PXG who had uncomplicated phacoemulsification and posterior chamber intraocular lens implantation surgery were included to our prospective study. Central corneal thickness (CCT), anterior chamber depth (ACD) and axial length (AL) were measured with an optical biometer preoperatively and at 1 month postoperatively. The pre- and postoperative values of intraocular pressure (IOP) and the anterior segment parameters and the differences between POAG and PXG were compared statistically by paired t, independent t and chi-square tests.

**Results:**

The mean values of preoperative CCT (p=0.042) and ACD (p=0.012) were significantly lower in the PXG than in the POAG group. In the PXG group, IOP decreased (p=0.001) but CCT (p=0.03) and ACD (p=0.001) increased significantly postoperatively; AL did not change significantly. In the POAG group, IOP decreased (p=0.01) and ACD (p=0.004) increased significantly postoperatively, while AL and CCT did not change significantly. There were no significant differences in the pre- to postoperative changes in IOP (p=0.76), AL (p=0.44) and CCT (p=0.52) values between the two groups. However, the postoperative increase in ACD was larger in the PXG group (p=0.03).

**Conclusion:**

Cataract surgery may cause some changes in IOP and anterior segment parameters like ACD and CCT postoperatively in eyes with POAG and PXG, and these changes may differ between eyes with PXG and POAG.

## INTRODUCTION

Cataract surgery lowers intraocular pressure (IOP) and reduces the need for anti-glaucomatous drugs, especially in patients with lens-induced glaucoma, angle-closure glaucoma and pseudoexfoliation glaucoma (PXG).^1,2,3,4,5,6^ Hayashi et al.^3^ found that cataract surgery lowered IOP in primary open-angle glaucoma (POAG) and angle-closure glaucoma, and reported a greater decrease in cases of narrow-angle glaucoma. The reduction in IOP is the result of increased anterior chamber depth (ACD) and widening of the iridocorneal angle following cataract surgery.^2,6,7,8,9,10^

Noncontact optical biometers use diode lasers and low-coherence reflectometry to allow the measurement of anterior segment parameters such as central corneal thickness (CCT), ACD, lens thickness and axial length (AL).^11,12,13^ The aim of this study was to determine the early effects of cataract surgery on IOP and anterior segment parameters such as CCT, ACD and AL measured at postoperative 1 month by optical biometry (Haag-Streit LENSTAR® LS 900 Optical Biometer, Switzerland) in POAG and PXG patients and to compare these effects between the two different types of glaucoma.

## MATERIALS AND METHODS

Twenty-five eyes of 25 patients with POAG and 29 eyes of 29 patients with PXG who underwent phacoemulsification and posterior chamber intraocular lens implantation in our hospital between September 2013 and December 2014 were included in this prospective study. The study was approved by the Ankara Numune Training and Research Hospital Ethics Committee and informed consent was obtained from all patients.

Pre- and postoperatively all patients underwent corrected visual acuity assessment using the Snellen chart, anterior and posterior segment examination, IOP measurement by Goldmann applanation tonometry, preoperative iridocorneal gonioscopy using a Goldmann three-mirror lens, optic disc and retinal nerve fiber layer evaluation by spectral-domain optical coherence tomography (OCT) and CCT measurement by ultrasonic pachymetry.

The exclusion criteria of the study were as follows: age less than 40 years old; presence of an active intraocular infection; previous ocular surgery or ocular trauma; history of uveitis; and glaucoma types other than PXG and POAG. Patients with diabetes mellitus were excluded due to possible effects on lens thickness. Furthermore, patients exhibiting widespread pterygium, leukoma, nebula, keratoconus or other corneal degeneration and dystrophies of the cornea on ophthalmologic examination, patients with elevated IOP or other glaucomatous findings refractory to medical treatment, and patients with any intra- or postoperative complications of the phacoemulsification procedure were not included in the study.

Patients over 40 years old were included in the study. Other inclusion criteria for POAG patients were as follows: being followed under anti-glaucomatous medical treatment in our glaucoma clinic; unmedicated IOP ≥22 mmHg; grade III-IV open iridocorneal angle according to the Shaffer classification; absence of glaucomatous findings at the optic disc (e.g. cup-to-disc ratio ≥0.3, localized neuroretinal rim notching, peripapillary choroidal atrophy, and splinter hemorrhage); and absence of glaucomatous visual field findings such as nasal step, arcuate scotoma or Seidel’s scotoma in the patient’s medical records. In addition to the inclusion criteria stated for POAG patients, the presence of pseudoexfoliation material at the pupillary margin and/or the lens surface was required for PXG patients. PXG patients with Shaffer grade I and II were not included in the study.

In addition to the detailed ophthalmologic examination, CCT, AL and ACD measurements were performed by optic biometry preoperatively and at the first postoperative month by the same experienced physician (K.T.).

The surgery was performed under topical anesthesia (Alcaine 0.5% ophthalmic solution, Alcon) beginning with a 2.8 mm clear corneal incision in the superotemporal quadrant, followed by phacoemulsification with torsional phaco technology (Infiniti, Alcon Laboratories Inc.) and single-piece hydrophobic posterior chamber IOL implantation (AcrySof® SA 30AL, Alcon), concluding with an intracameral cefuroxime (1 mg/0.1 ml) injection. In the postoperative period, patients received topical moxifloxacin hydrochloride (Vigamox® 0.5% ophthalmic solution, Alcon) four times a day for one week and topical prednisolone acetate (Predforte® 1% ophthalmic solution, Allergan) four times a day for one month. The t test, independent samples t test and chi-square test were used in statistical analyses.

## RESULTS

The POAG group consisted of 25 (12 female and 13 male) patients with a mean age of 64.9±8.5 (range, 46-75) years; the PXG group consisted of 29 (15 female and 14 male) patients with a mean age of 69.6±6.3 (range, 60-77) years. The gender distributions of the groups were statistically equivalent, but the PXG group was statistically older than the POAG group (p=0.32 and p=0.043, respectively) (Table 1).

Preoperative IOL, AL, CCT and ACD values are summarized in Table 2. All patients’ glaucoma was medically controlled; therefore there was no significant difference between groups in terms of IOL achieved with medication (p=0.84). Preoperative number of anti-glaucomatous drugs used was 1.15±0.5 in the POAG group and 1.4±0.5 in the PXG group. Preoperative mean CCT and ACD were found to be significantly lower in PXG eyes compared with POAG eyes (p=0.04 and p=0.01, respectively); no significant difference was detected in AL between the groups (p=0.21) (Table 2).

Pre- and postoperative IOP and other anterior segment parameter values, as well as the postoperative changes in these parameters are presented in Table 3. In PXG eyes, IOP decreased postoperatively (p=0.001) while CCT and ACD values increased (p=0.029 and p=0.001, respectively). There was no significant difference in AL (p=0.44). In POAG eyes, IOP decreased postoperatively (p=0.01) while ACD values increased (p=0.04). However, no difference was observed in CCT or AL values (p=0.31 and p=0.42, respectively). The number of anti-glaucomatous drugs used postoperatively was 1.1±0.6 in the POAG group and 1.2±0.6 in the PXG group.

Comparison of the surgically induced anterior segment changes between PXG and POAG eyes revealed no significant differences in IOP (p=0.76), AL (p=0.44) or CCT (p=0.52) changes, whereas the postoperative increase in ACD was significantly greater in PXG eyes (p=0.03) (Table 3).

## DISCUSSION

In both glaucomatous and normal eyes, cataract surgery increases ACD and widens the iridocorneal angle, thereby decreasing IOP.^1,2,3,4,5,6,14^ Huang et al.^14^ used anterior segment OCT to investigate ACD and anterior chamber angle in angle-closure glaucoma and open-angle glaucoma patients after cataract surgery and found that cases with narrow angles in particular showed larger reduction in IOP. The current study included POAG and open-angle PXG patients in order to determine the effects of cataract surgery on IOP and anterior segment parameters such as CCT, ACD and AL in these two groups of patients with open angles.

Cataract surgery is the main surgical treatment method for primary angle-closure glaucoma (PACG) patients and is preferred by many practitioners over trabeculectomy.^7,15^ Zhao et al.^7^ analyzed 85 PACG patients by Pentacam imaging 3 months after cataract surgery and found a significant IOP reduction as well as increased anterior chamber volume and wider anterior chamber angle.

Although these effects may be more pronounced in patients with angle-closure glaucoma, cataract surgery may also lead to a drop in IOP and changes in anterior segment parameters in patients with open-angle glaucoma^.3^ Because all of the subjects in our study had open-angle glaucoma, a secondary aim of our study was to evaluate the effect of another variable, pseudoexfoliation, on these parameters.

Dooley et al.^9^ used the Pentacam to evaluate the effects of uncomplicated cataract surgery on anterior segment morphology in normal, nonglaucomatous eyes and observed that IOP decreased by an average of 3.2 mmHg while anterior chamber angle, depth and volume increased postoperatively. They also showed that the preoperative IOP/ACD ratio fell postoperatively in proportion to the decrease in IOP.^9^ We also observed significant increases in ACD after cataract surgery in both POAG and PXG patients, with a greater increase in PXG patients. Ciliary zonular laxity is a probable cause of the shallower anterior chamber in PXG patients.^16^ Doganay et al.^17^ showed that PXG patients have relatively shallower anterior chambers than control subjects. Consistent with the literature, in the current study the preoperative ACD was significantly shallower in PXG patients than POAG patients. Furthermore, the postoperative increase in ACD was larger in PXG patients compared to POAG patients. Although IOP declined significantly in both of our study groups, there was no significant difference between the groups. In addition, we detected no significant pre- to postoperative changes in CCT. As in the current study, Dooley et al.^9^ examined CCT changes in the sixth week after cataract surgery and also found no significant differences.

Our results revealed no significant differences between pre- and postoperative AL. In contrast, Seok et al.^18^ found that AL increased significantly after cataract surgery. In a Turkish study, Bilak et al.^19^ reported a significant decrease in AL values at 1 month after cataract surgery in healthy subjects. AL may also decrease after trabeculectomy.^20^ Brown^20^ demonstrated that AL values decreased in proportion to the fall in IOP following trabeculectomy. Unlike both of those studies, we detected no significant change in AL values postoperatively.

## CONCLUSION

Uncomplicated phacoemulsification with posterior chamber IOL implantation surgery in POAG and PXG patients can lead to significant changes such as lower IOP and greater ACD. To the best of our knowledge, there are no reports in the literature comparing the effects of cataract surgery on anterior segment parameters measured by optical biometry in patients with POAG and PXG. The larger postoperative increase in the ACD values of PXG patients is likely related to ciliary zonular laxity in these patients. Future studies are being planned to include larger patient numbers and a wider variety of glaucoma types and to utilize different imaging methods like anterior segment OCT and Pentacam.

## Ethics

Ethics Committee Approval: Our presentation was approved by Ethics Committee of Ankara Numune Training and Research Hospital, Informed Consent: The study was approved by the Ankara Numune Training and Research Hospital ethics committee and informed consent was obtained from all patients.

Peer-review: Externally and internally peer-reviewed.

## Figures and Tables

**Table 1:**
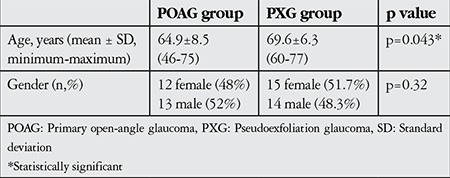
Demographic characteristics of the patients

**Table 2:**
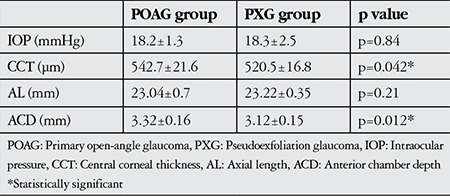
Preoperative mean intraocular pressure, central corneal thickness, axial length and anterior chamber depth values

**Table 3:**
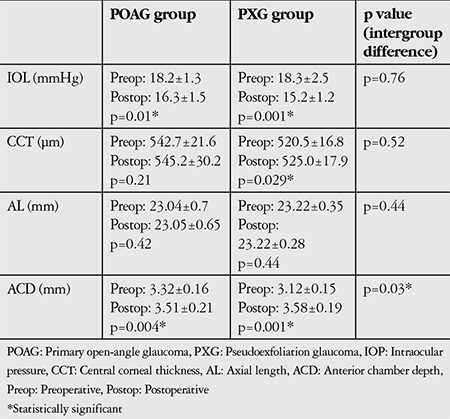
Preoperative and 1 month postoperative intraocular pressure, central corneal thickness, axial length and anterior chamber depth values
